# SPIRITUAL NEEDS OF ELDERLY IN CHINESE FAITH-BASED NURSING HOME: ADMINISTRATOR VIEWS

**DOI:** 10.1093/geroni/igad104.3599

**Published:** 2023-12-21

**Authors:** Lin Jiang, David Hodge, Robin Bonifas, Fei Sun

**Affiliations:** The University of Texas Rio Grande Valley, Edinburg, Texas, United States; Arizona State University, Phoenix, Arizona, United States; Indiana State University, Terre Haute, Indiana, United States; Michigan State University, East Lansing, Michigan, United States

## Abstract

**Objectives:**

Recognizing and addressing the spiritual needs of elderly individuals constitutes a crucial facet of comprehensive caregiving. Despite China’s immense aging population, research on the spiritual needs of Chinese seniors remains limited. This study aimed to: 1) delineate prevalent spiritual needs among Chinese nursing home residents, including those with dementia; 2) elucidate the method employed by staff to identify these needs in non-verbal residents; and 3) expound on the strategies utilized by staff to fulfill these identified needs.

**Methods:**

In this qualitative exploration, semi-structured interviews were conducted with 21 administrators from faith-based nursing homes, chosen for their potential concentration of residents with dementia. Encompassing establishments across 14 Chinese provinces, interviews were conducted in the participants’ native language, subsequently translated into English. Utilizing a constant comparative approach, the collected data underwent analysis to uncover recurring themes.

**Results:**

The analysis yielded five interconnected themes concerning the spiritual needs of older adults: expression of faith, reception of love and care, familial interaction, social engagement, and participation in activities. For individuals with dementia unable to articulate their needs, staff employed careful observation and communication with family members as means of identification. In response to identified needs, staff harnessed local resources and personalized services whenever feasible.

**Discussion:**

The findings offer practical insights for holistic caregivers catering to the spiritual needs of Chinese elderly. This study fills an existing gap, aiding in the provision of culturally sensitive care that addresses the spiritual aspects of aging Chinese individuals.

